# Retraction Note: Contrasting the complexity of the climate of the past 122,000 years and recent 2000 years

**DOI:** 10.1038/s41598-020-61685-w

**Published:** 2020-03-19

**Authors:** Zhi-Gang Shao

**Affiliations:** Guangdong Provincial Key Laboratory of Quantum Engineering and Quantum Materials, SPTE, South China Normal University, Guangzhou, 510006 China

Retraction of: *Scientific Reports* 10.1038/s41598-017-04584-x, published online 23 June 2017

The Editors have retracted this article.

After publication it was brought to the Editors’ attention [1] that it is not correct to compare values of sample entropy that were obtained from time series having vastly different lengths and sampling times, as was done in this study. The increased likelihood for nonstationarity in the longer series as well as different sampling times both significantly affect the probability of the occurrence of patterns in the data, and thus lead to inherently irrelevant comparisons of obtained sample entropy values. This means that the observed differences in sample entropy between the two time series in this study cannot be attributed to actual changes in climatic complexity. The Editors therefore no longer have confidence in the conclusions of this article.

Zhi-Gang Shao disagrees with the retraction.
